# Incidence of neutrophil extracellular traps (NETs) in different membrane oxygenators: pilot in vitro experiments in commercially available coated membranes

**DOI:** 10.1007/s10047-024-01486-4

**Published:** 2025-01-08

**Authors:** M. Foltan, D. Dinh, M. Gruber, T. Müller, C. Hart, L. Krenkel, C. Schmid, K. Lehle

**Affiliations:** 1 Department for Cardiac, Thoracic and Cardiovascular Surgery, University Hospital Regensburg, Regensburg, Germany; 2 Department for Anaesthesiology, University Hospital Regensburg, Regensburg, Germany; 3 Department for Internal Medicine II, University Hospital Regensburg, Regensburg, Germany; 4 Department for Internal Medicine III, University Hospital Regensburg, Regensburg, Germany; 5 Regensburg Center of Biomedical Engineering, University and OTH Regensburg, Regensburg, Germany

**Keywords:** NETosis, NETs, ECMO, Membrane oxygenators, Neutrophil granulocytes

## Abstract

Neutrophil extracellular traps (NETs) were detected in blood samples and in cellular deposits of oxygenator membranes during extracorporeal membrane oxygenation (ECMO) therapy and may be responsible for thrombogenesis. The aim was to evaluate the effect of the base material of gas fiber (GF, polymethylpentene) and heat exchange (HE) membranes and different antithrombogenic coatings on isolated granulocytes from healthy volunteers under static culture conditions. Contact of granulocytes with membranes from different ECMO oxygenators (with different surface coatings) and uncoated-GFs allowed detection of adherent cells and NETotic nuclear structures (normal, swollen, ruptured) using nuclear staining. Flow cytometry was used to identify cell activation (CD11b/CD62L, oxidative burst) of non-adherent cells. Uncoated-GFs were used as a reference. Within 3 h, granulocytes adhered to the same extent on all surfaces. In contrast, the ratio of normal to NETotic cells was significantly higher for uncoated-GFs (56–83%) compared to all coated GFs (34–72%) (*p* < 0.001) with no difference between the coatings. After material contact, non-adherent cells remained vital with unchanged oxidative burst function and the proportion of activated cells remained low. The expression of activation markers was independent of the origin of the GF material. In conclusion, the polymethylpentene surfaces of the GFs already induce NET formation. Antithrombogenic coatings can already reduce the proportion of NETotic nuclei. However, it cannot be ruled out that NET formation can induce thrombotic events. Therefore, new surfaces or coatings are required for future ECMO systems and long-term implantable artificial lungs.

**Supplementary Information:**

The online version contains supplementary material available at 10.1007/s10047-024-01486-4.

## Introduction

Extracorporeal membrane oxygenation (ECMO) is a life-saving treatment option for patients in severe acute but potentially reversible cardiopulmonary failure [1]. The replacement of the ECMO system often represents an acute life-threatening situation for the patient. Up to now, the causes of thrombotic ECMO complications, range between 3 and 26% [2–4], are still largely unknown.

These ECMO complications lead to a reduction in gas exchange capacity [5, 6]. The thrombi consist of fibrin strands with embedded platelets and erythrocytes, which, however, differ in their appearance depending on their localization within the oxygenator and the anticoagulation used [7]. Adherent leukocytes were also found in platelet deposits, indicating the involvement of inflammatory reactions in thrombogenesis in the oxygenator [8, 9]. Further investigations include the identification of von Willebrand factor as a thrombus component to identify possible mechanisms of thrombogenesis [10].

The involvement of polymorphonuclear neutrophil granulocytes (PMNs) in this process [11] can contribute to pathological thromboses or “immunothromboses” through the release of neutrophil extracellular traps (NETs) [12]. NETs are network structures of extracellular fibres that consist mainly of the DNA of PMNs and are covered with granular proteins (e.g. myeloperoxidase (MPO), neutrophil elastase (NE)). Pathogens and platelets can bind to these fibres, which can lead to inactivation of pathogens and thrombus formation [13]. NETs could be detected on biocompatible materials with hydrophobic surfaces [14] and clinically used oxygenators [15] by extracellular DNA, NE and citrullinated histones. In addition, the aim of the present study was to investigate material-induced NETosis as a possible mechanism for thrombus formation in the ECMO oxygenator. As there are obviously oxygenator type-dependent differences in leukocyte adhesion [8] and thus possibly also in NETosis formation, the gas fibres of commercially available oxygenators should be examined for a NETosis-inducing effect. The gas fibres from all oxygenators consisted of polymethylpentene (PMP) but differed in the type of antithrombogenic surface coatings.

## Materials and methods

Details of materials and methods see supplementary material.

### Test material

Test material consisted of gas exchange fibres (GF) and heat exchange membranes (HE) from various commercially available ECMO oxygenators with different non-thrombotic surface coatings [PLS, Bioline® coating (Getinge, Goteborg, Sweden); Hilite 7000LT, X.ELLENCE® coating (Fresenius, Bad Homburg, Germany); Nautilus, Balance® coating (Medtronic, Dublin, Ireland); EOS, PH.I.S.I.O® coating (LivaNova, London, UK); Table S1]. Uncoated-GFs membrane mats (reference material) were kindly provided by Getinge. Uncoated-GFs and GFs from all oxygenator types consisted of PMP. HEs were made of different material (Table S1). One naïve oxygenator of each manufacturer was analyzed.

For preparation of the test material a band saw was used to open the oxygenator housing. The GF/HE membrane core was sterile-packed. GF and HE membrane mats were separated without local assignment, cut to size (“samples”, length x width, 25 mm x 5 mm, 10 fibres connected via wrap threads), fixed in sterilized stainless steel inserts and placed in sterile Petri dishes (diameter, 35 mm; Thermo Fischer, Waltham, Massachusetts, USA) (Figure S1). The samples were coated with 3 mL HTP buffer + 30% AB serum (12 h, gentle movement, room temperature), dried, and used for PMN adhesion/activation within 24 h.

Poly-L-lysine-coated (0.01%, Sigma-Aldrich, St. Louis, USA) glass slides served as control samples to prove PMN adhesion and induction of NETosis.

### Isolation of granulocytes

Granulocytes were isolated from lithium-heparin (16 IU/mL) anticoagulated blood samples of healthy volunteers (*n* = 6) using double density gradient centrifugation. Isolated PMNs from each volunteer were incubated with all test materials (one sample from each oxygenator type) in six independent experiments. Blood donors gave informed consent for inclusion before participation in the study. The study protocol was approved by the Ethics Committee of the University of Regensburg (vote no. 16-101-0322).

The cell density was counted using an analysis system (CASY, OMNI Life Science, Bremen, Germany) and adjusted to 2.5 * 10^5^ cells / mL with the resuspension buffer.

For each oxygenator type, a negative control (adhesion of granulocytes and incubation in buffer) and a positive control (adhesion of granulocytes and incubation in 200 nM phorbol-12-myristate-13-acetate (PMA)) were always analysed to identify NETotic nuclei [16].

### Contact of granulocytes with test materials

Dried samples were wetted with HTP-BSA-Ca/Mg (3 mL, 10 min, RT). After aspiration, 1 mL of freshly HTP-BSA-Ca/Mg was transferred below the clamped samples and 2 mL of the cell suspension was deposited on top of the samples. Petri dishes were capped and incubation was carried out for 3 h at 37 °C with gentle movement (shaking incubator, Kuhner Kelvin, Basel, Switzerland). The surfaces of the samples were analyzed for granulocyte adhesion and NET formation. The supernatants were collected to detect material-induced cell activation via flow cytometry (FACS).

### Granulocyte adhesion and NET formation: immunolabeling

After washing twice with HTP buffer, the samples were stained with 4′,6-diamidino-2-phenylindole (DAPI, 500 ng/mL) (60 min, RT, dark), washed with 2 mL HTP buffer and covered with Fluoromount-GTM mounting medium (Thermo Fisher). Adherent cells were fixed with paraformaldehyde (4% in PBS, 3 mL, 10 min, room temperature). The samples were located between two coverslips and cured in the refrigerator for 3 days. DAPI-stained nuclei were visualized using a fluorescence microscope (BZ-8100E Keyence, Neu-Isenburg, Germany) with an integrated camera system (magnification, 16x to 80x).

Adherent granulocytes (on glass slides and GF samples) were stimulated with PMA (200 nM) for 30 min after a 3 h adhesion phase (positive control). Similar incubation with HTP-BSA-Ca/Mg was used as a negative control.

DAPI-staining allowed the quantification of cell density and the differentiation of nuclear morphologies to identify NETs [16]. Granulocytes without morphological changes in the form of nuclear deformations or protrusions are termed “normal”. Swollen nuclei and ruptured nuclei with ejected DNA were identified as NETs. A cell was considered swollen if its original outline was clearly deformed, with expansions and bulges that were more than half the size of the nucleus. The ratio of NETotic nuclei relative to the total cell number was examined.

ImageJ, an open platform for scientific image analysis was used for processing and analysing the fluorescence microscope images. The cell count was determined for each sample (detection area, 4.1 mm^2^; magnification, 40x) using the previously validated (Figure S2) cell automatically counting function (Figure S3). Ten randomly selected non-overlapping microscopic images from each sample (GF surfaces without wrap threads) were evaluated. In total, 300 microscopic images (10 images/sample, 6 blood donors, 5 different GF-coatings) were used for detection of cell counts. Additional 180 microscopic images (6 images/sample, 6 blood donors, 5 different GF-coatings) were recorded (magnification, 80x) and NETotic nuclei were manually counted by an experienced specialist. The ratio of NETotic nuclei (Figure S4) relative to the automatically detected cell count was determined per defined area of interest (2.05 mm^2^; Figure S4A–E).

### NET degradation using DNAse treatment

DNAse is essential for NET degradation and was used to identify NETs under in vitro conditions [17]. Therefore, PMA-stimulated (and non-stimulated) granulocytes (seeded on poly-L-lysine-coated glass slides) were treated with DNase-1 (deoxyribonuclease I, Roche, Merck, Darmstadt, Germany) (10 µg/mL, in HTP-BSA-Ca/Mg). The removal of extracellular NET-structures and the retention of non-altered nuclei indicated the existence of NETs.

### Material-induced activation of granulocytes: FACS analysis

Non-adherent cells in the supernatants were collected to detect material-induced cell activation via flow cytometry (FACS). This included the formation of ROS (reactive oxygen species) (oxidative burst) and the expression of activation markers (CD11b, CD62L (L-Selectin)). Details of sample preparation and gating strategy see Figure S5 and supplementary material.

### Statistical analysis

The SigmaPlot 13.0 software programme (Systat, Erkrath) was used for statistical analysis of the experiments. The quantitative comparison of the neutrophil accumulations and the NETotic changes on the differently coated ECMO membranes was carried out using one-factorial ANOVA due to the non-normally distributed values. Statistical significance was assumed for a *p* value < 0.05.

## Results

### Granulocyte adhesion and NET formation on different ECMO fibers

**Fig. 1:**
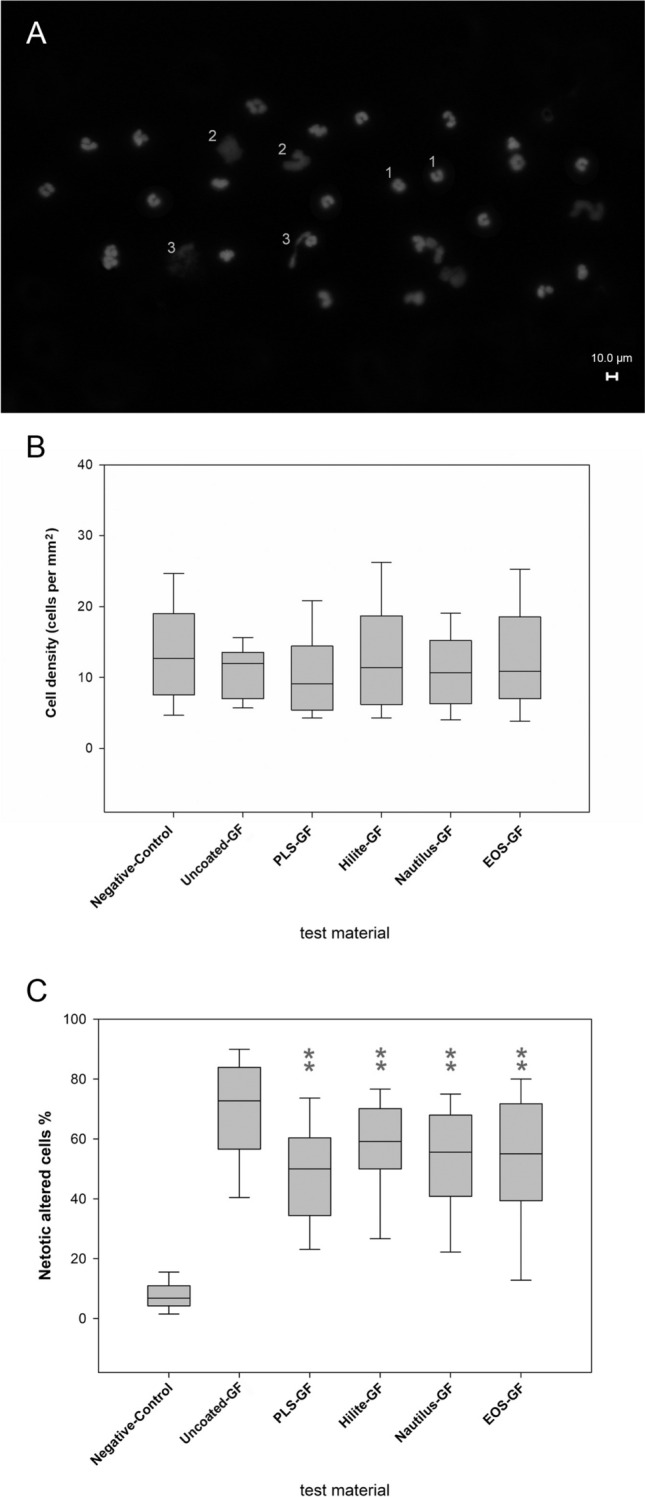
Contact of isolated granulocytes with surfaces of different GF-types (test material) resulted in granulocyte adhesion and NET forming. **A** Representative sample of adherent and DAPI-stained granulocytes on a PLS-GF with normal (1) and NETotic nuclei (swollen nuclei (2), ejection of DNA (3)), (80x magnification). **B** Quantification of cell density of adhering granulocytes on GFs from different oxygenator types (300 randomly selected microscopic images from 10 gas fibres of each sample). Cells were evenly distributed and cell density was comparable (*p* = 0.230). **C** Quantification of the ratio of NETotic to all analysed nuclei (215 non-overlapping microscopic images). The percentage of NETotically altered cells was significantly higher in the uncoated-GF compared to all other GFs. (**p < 0.01). Uncoated and coated GFs exhibit a significantly higher number of NETotically altered granulocytes compared to the negative control on glass surfaces (*p* < 0.01). Data are median (IQR). Statistics, one-way ANOVA

Independent of the origin of the GFs, granulocytes adhered on the surface of all GFs (Figure S4). The cells were evenly distributed along the GF (Fig. 1A). The median cell density (IQR) was 10 [6–16] cell nuclei per mm^2^ and was independent of the GF types (coatings) (Fig. 1B). There was no significant difference in the cell density comparing uncoated-GF with all other GFs (*p* = 0.230).

Figure 1A shows a representative image of different nuclear morphologies (normal, swollen nuclei and ejected DNA) after a 3 h incubation of isolated granulocytes with a PLS-GF sample. The proportion of NETotic nuclear structures was significantly higher on the uncoated-GF samples compared to all coated samples (*p* < 0.001) (Fig. 1C). There was no difference between the coated samples (Fig. 1C). In contrast, the proportion of NETotically altered granulocytes was significantly higher on any type of GFs compared to the negative control with PLL-coated glass surfaces (Fig. 1C).

**Fig. 2:**
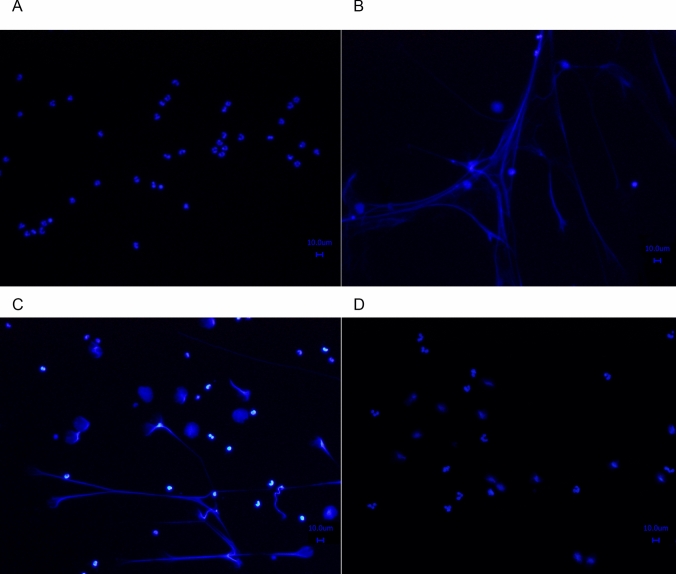
NET formation on PLS-GFs. Adherent granulocytes on a PLS-GF (80x magnification) after 3 h incubation with **A** buffer (negative control) and **B** PMA (positive control). PMA induced NET formation presenting different nuclear morphologies (predominantly completely ejected DNA). **C** PMA-treated granulocytes on a PLS-GF formed NETs that were removed after DNAse I treatment (**D**)

Treatment of adherent granulocytes with PMA proved their ability to induce NETosis (Fig. 2). While the incubation of adherent granulocytes on PLS-GFs with buffer (negative control, Fig. 2A) had no effect on the nuclear morphology, the treatment with PMA (positive control, Fig. 2B) induced expulsion of DNA. Finally, PMA treatment resulted in formation with more than 90% NETotic nuclei. The additional treatement of NETotic nuclei with DNAse I resulted in a complete removal of extracellular DNA (Fig. 2C and D). Normal nuclei remained adherent on the surface of the PLS-GF.

ECMO oxygenators not only consisted of GFs but also heat exchanger membranes (HE). HE consisted of different materials (Table S1) with a high transparency (e.g. PLS-HE made of transparent PUR). The physical properties prevented the detection and quantification of DAPI-stained adherent granulocytes. Therefore, these materials were excluded from adhesion experiments. However, material-induced activation was analyzed using FACS analysis (see below).

### Material-induced activation of granulocytes

**Fig. 3:**
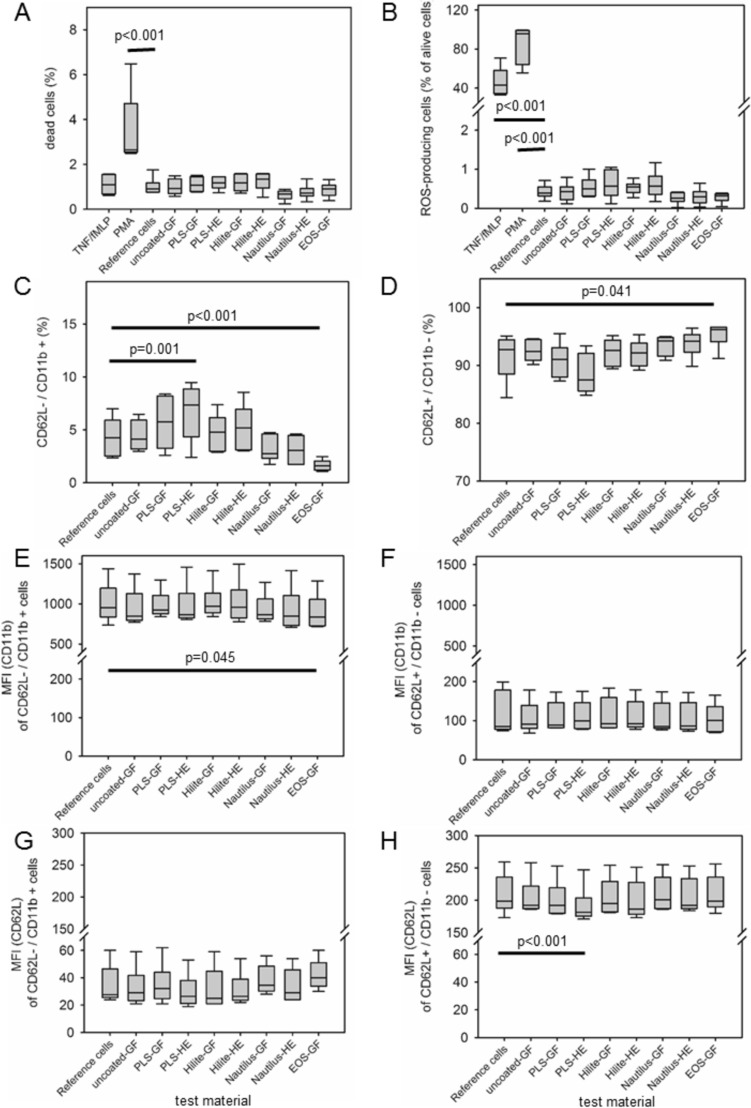
Contact of isolated granulocytes with test materials did not affect viability (**A**), did not induce ROS production (**B**), only marginally activate the cells (**C**, **D**) without alteration of expression of CD11b and CD62L (**E**–**H**). Granulocytes in the supernatant of the adhesion experiments (*n* = 6) were vital (> 98%) (**A**), and showed no ROS production (**B**). The proportion of CD62L-/CD11b + cells (= activated cells) (**C**) and CD62L + /CD11b− cells (= non-activated cells) (**D**) remained below 10%. The median fluorescence intensity (MFI) of CD11b (**E**, **F**) and CD62L (**G**, **H**) remained at the level of reference (not-stimulated cells without contact to test materials). TNF/fMLP and PMA, stimulation of reference cells with TNF/fMLP or PMA as described in the method section. Statistics, two-way ANOVA on variance. (Gas fibre membranes (GF), heat exchanger membranes (HE))

The contact of isolated granulocytes with artificial surfaces such as GFs and HEs can trigger an oxidative burst and an upregulation of activation markers (CD11b, CD62L) of non-adherent cells in the supernatant. Reference cells (without contact to test material) showed a significant increase in the proportion of dead cells after incubation with PMA, while TNF/fMLP had no effect (Fig. 3A) on the viability. Incubation of the vital cells with PMA or TNF/fMLP led to a complete (96 [64–99] %) or partial (43 [34–58] %) provocation of the cells to trigger an oxidative burst (Fig. 3B). In contrast, isolated granulocytes that were in contact with the coated or uncoated samples for 3 h showed no significant difference in the ROS production compared to the reference cells (Fig. 3B). Furthermore, contact of granulocytes with the test materials did not lead to any additional alteration of the activation markers CD62L and CD11b (Fig. 3C). While the proportion of CD62L + /CD11b− cells (non-activated) (Fig. 3D) remained constant, the proportion of CD62L-/CD11 + cells (activated) (Fig. 3C) showed marginal alterations. Contact of the cells with the PLS heat exchanger samples (PLS-HE) resulted in a significant increase in the proportion of activated cells (*p* = 0.001), while the EOS-GF samples settled down (*p* < 0.001) (Fig. 3D). No significant difference was detectable between gas fibre samples and heat exchanger samples. In addition, the median fluorescence intensity of cells in the supernatant remained unchanged and at the level of the reference cells (Fig. 3E–H).

## Discussion

Clinical evaluation reports have shown that the surface of gas exchange membranes within used ECMO oxygenators were covered with leukocytes 8–10, 12, 18. Our in vitro study supported these clinical observations, as we were able to confirm that the surface of the non-used PMP gas fibers (independent of the surface coatings) caused remarkable adhesion of granulocytes. However, the contact of isolated granulocytes with uncoated-GFs induced a higher frequency of NETs compared to all analyzed coated PMP surface. In contrast, phenotype (expression of CD11b and CD62L) and oxidative burst function of floating granulocytes remained unchanged after contact with GFs and HEs from commercially available ECMO oxygenators.

The type of antithrombotic surface coating of GFs did not affect granulocyte adhesion under static conditions. Already the surface properties of the originally uncoated PMP membrane form the manufacturer (OxyPlus 90/200, Membrana/3 M, Obernburg, Germany) induced cell adhesion. Wiegmann et al. [19] demonstrated complete endothelialization of heparin/albumin coated PMP-GFs under static and dynamic conditions. This was in agreement with LaIuppa et al. [20] who demonstrated adhesion and expansion of peripheral blood mononuclear cells and CD34 + progenitor cells on PMP surfaces. Cell adhesion was improved by serum-coating. We also precoated the PMP surfaces with 30% human serum to simulate in vivo conditions. Immediately upon exposure of a biomaterial to blood, a diverse repertoire of plasma proteins adsorbed to the surface within seconds. These proteins include albumin, fibrinogen, high-molecular-weight kininogen, coagulation factor XI and FXII/XIIa, plasma prekallikrein, immunoglobulins (IgG, IgM), complement proteins, vitronectin, fibronectin, apolipoproteins and von Willebrand factor. The composition of the protein layer depends on specific properties of the surface and may vary over time [21, 22]. This is a nidus for triggering activation of several cellular and blood-based proteolytic cascades [22]. The inflammatory responses included material-induced activation of circulating granulocytes and increased adhesion to the artificial surface [23]. Triggers for leukocyte activation on materials mainly are adsorbed proteins (mainly fibrinogen, fibronectin, iC3b) and adherent platelets. Upregulation of CD11b, loss of L-selectin, release of reactive oxygen species (oxidative burst), NE, cathepsin G or IL-8 are typical markers of activation [24]. Isolated granulocytes were non-activated as shown with low oxidative burst as well as moderate expression of CD11b and high expression of CD62L. The contact of isolated granulocytes with uncoated and coated PMP surfaces resulted in minimal activation of the granulocytes. The proportion of CD11b−/CD62L + cells (as well as their median fluorescence intensity (Fig. 3) remained unchanged after contact with test samples for 3 h. However, the moderate expression of CD11b seems to be high enough to result in cell adhesion onto PMP surfaces. Cell adhesion was mainly mediated through surface-bound iC3b and fibrinogen that bind to CD11b/CD18 on leukocytes [25, 26]. Increased expression of CD11b was also shown in in vivo studies including the surface of stents [27], oxygenators [28] and hemodialysis membranes [29].

The primary goal of passive and bioactive surface coatings is to reduce the thrombogenicity of the artificial material [30]. Different coating strategies were used for different ECMO oxygenators: Grafted polymer brushes (Nautilus), zwitterionic coatings (Phosphorylcholine, EOS), and heparin-coatings (PLS, Hilite) reduced fibrinogen and platelet adhesion, however, clinical efficacy is lacking [30]. However, despite different coatings with different mechanisms of action in the clinical usage there was no prevalence for a specific ECMO system that reduced or prevented the development of a coagulation disorders that resulted in device-induced thrombosis (e.g. oxygenator thrombosis) [2, 3]. Furthermore, the importance of leukocytes in thrombogenesis is often neglected. As shown in the present study, the inflammatory reactions appear to be independent of the surface coatings and could therefore be primarily responsible for the formation of thrombi in the oxygenators. Only one clinical study analyzed the surface coverage of different GFs. Dropco et al. presented a significantly higher cellular accumulation on PLS-GFs compared to Hilite-GFs of clinically used oxygenators. However, randomly selected samples from the oxygenators were used without paying attention to the location of the samples in the oxygenator or the presence of overlying thrombi. In addition, there was a clear disproportion in the number of samples for the individual oxygenator types (Hilite, *n* = 7; PLS, *n* = 26). This makes type-dependent leukocyte adhesion questionable.

Adhesion of granulocytes on non-coated and coated PMP surfaces and cultivation for 3 h in serum-free medium resulted in the alteration of the nuclear morphology. The changes manifested in a swelling of the cell nucleus and an expulsion of DNA. Finally, 34 to 84% of the adherent granulocytes presented altered nuclear morphologies. Both morphologies were detected during NET formation after stimulation of adherent granulocytes with PMA at different time points—nuclear swelling was defined as a precursor form of NET forming [16]. These compounds are highly thrombogenic [31] and have been identified in thrombi from coronary arteries [32] failed stents [33], and ECMO oxygenators [34]. Recently, Sperling and Maitz found more NET-compounds (DNA, citrullinated histones, NE) on hydrophobic surfaces (Teflon AF, self-assembled monolayers of methyl terminated alkanethiols) compared to hydrophilic surfaces (PEMA, poly(ethylene-alt-maleic anhydride)). They suggested that NETs may also contribute to biomaterial-induced thrombogenicity [14]. Uncoated as well as all coated PMP surfaces were hydrophilic and presented improved biocompatibility [35]. Material-induced NET formation on PMP surfaces was not analyzed so far. In the present study, particularly striking was the high proportion of NETotic nuclei on all test samples ranging between 34 and 83%. The highest NET formation was detected on uncoated PMP surfaces, while antithrombotic surface coatings reduced NET formation. However, the high rate of NETosis must be viewed with caution. The majority of altered nuclei were rounded up and enlarged and were characterized as a precursor state of NETs [16]. Less than 10% of the nuclei were without a nuclear membrane. However, if the adherent cells were treated with PMA for 3 h, a rupture of almost all nuclei was observed. The subsequent treatment with DNAse disrupted the DNA backbone of NETs as already shown in complete removal of expulsed DNA. The extent to which the altered nuclear morphologies are NET precursors cannot be conclusively answered. Since we used isolated granulocytes the preparation protocol—in particular centrifugation steps—could also lead to altered adhesion or reduced functionality of the cells [36] resulting in a reduced viability. In clinical studies, NETs were detected in the plasma of ECMO patients, but their origin could not be clearly traced back to ECMO support [37, 38]. However, decondensed nuclei that filled the entire cell were not only detected in the process of NETosis [16] but also on the surface of PMP-GF from a PLS membrane lung at the end of an ECMO therapy [15]. However, systematic analysis of NET-structures within different oxygenators of clinically used ECMO systems or from animal experiments failed so far. Both approaches could provide insights into how physiological factors like blood flow and inflammation affect NETosis. However, animal models with ECMO therapy usually use large animals (pigs) and are therefore very complex and expensive. Furthermore, clinical studies are always associated with various influencing factors (such as the underlying disease, gender, coagulation status) that aggravate a correlation of NET formation and thrombosis during ECMO.

This study has two limitations. This is an in vitro study that used isolated granulocytes from healthy volunteers. Both, isolation as well as cultivation protocol may affect cell viability.

## Conclusion

Contact of granulocytes with PMP surfaces resulted in the alteration of nuclear morphologies including the existence of decondensed as well as explored nuclei that may be indicators for material-induced NET forming. Antithrombogenic coatings can already reduce the proportion of NETotic nuclei. However, it cannot be ruled out that NET formation can induce thrombotic events. Further studies demanded the development of new surfaces or surface coatings not only to reduce thrombogenicity but also their NET-forming properties. This might also be an attractive strategy for alternative anticoagulation strategies.

## Supplementary Information

Below is the link to the electronic supplementary material.

Supplementary file1 (DOCX 3516 KB)
