# Supplementary material for: Incidence of neutrophil extracellular traps (NETs) in different membrane oxygenators: pilot in vitro experiments in commercially available coated membranes
Source: J Artif Organs. 2025 Jan 8;28(3):374–82. doi: 10.1007/s10047-024-01486-4 (PMC12373553; doi:10.1007/s10047-024-01486-4)
Supplement: Supplementary file 1 — Supplementary file1 (DOCX 3516 KB) [file 10047_2024_1486_MOESM1_ESM.docx]

**Electronic supplementary material**

**Incidence of Neutrophil Extracellular Traps (NETs) in different membrane oxygenators**

- Pilot in-vitro experiments in commercially available coated membranes -

Foltan M, Dinh D, Gruber M^1^, Müller T^2^, Hart C^3^, Krenkel L^4^, Schmid C, Lehle K

Department for Cardiac, Thoracic and Cardiovascular Surgery, University Hospital Regensburg

^1^ Department for Anaesthesiology, University Hospital Regensburg

^2^ Department for Internal Medicine II**,** University Hospital Regensburg

^3^ Department for Internal Medicine III, University Hospital Regensburg

^4^ Regensburg Center of Biomedical Engineering, University and OTH Regensburg

Table S1. Coatings and HE material of commercially available oxygenator membranes. All coated or uncoated GFs are made out of PMP

| **Oxygenator type**  **(manufacturer), surface area** | **Coating** | **Mode of operation** | **HE, material, surface area** |
| --- | --- | --- | --- |
| PLS (Getinge, Rastatt), 1.8 m^2^ | Bioline | Covalent bonds between heparin molecules and an albumin layer. | Polyurethane (PUR), 0.4 m^2^ |
| Hilite 7000 LT (Fresenius, Bad Homburg), 1.9 m^2^ | X.ELLENCE | Heparin covalently and ionically bound to immobilised albumin in several layers. | Polyethylene terephthalate (PET), 0.45 m^2^ |
| Nautilus (Medtronic, Meerbusch), 1.8 m^2^ | Balance | Polyethylene oxide (PEO), integration of sulphate and sulphonate groups. | Polyethylene terephthalate (PET), 0.3 m^2^ |
| EOS (LivaNova, Munich), 1.2 m^2^ | PH.I.S.I.O | Phosphorylcholin (PC) | Stainless steel, 0.14 m^2^ |
| PMP (Getinge, Rastatt) | Uncoated | - | - |

**Details of oxygenator coatings**

**Nautilus oxygenator membrane with balance coating**

The basis of the balance coating consists of the polymer polyethylene oxide (PEO). In this polymer coating, the functional layer is firmly bonded to the artificial ECMO surface via a primer. Sulphate and sulphonate groups are integrated into this hydrophilic functional layer to imitate the negative charge of the vascular endothelium. Research results show that negatively charged sulphonated polymers repel negatively charged thrombocytes [1] and inhibit thrombin by binding to antithrombin in a similar way to heparin [2][3]. An "insulating" water-like structure is established between the artificial ECMO surface and the patient's blood via the hydrophilic polymer molecules, which is intended to reduce protein deposits and cell adhesions [4].

**Hilite oxygenator membrane with X.ELLENCE coating**

In the biocompatible and haemocompatible X.ELLENCE coating, heparin is covalently and ionically bound to immobilised albumin in several layers. The multi-layer coating is stabilised by final cross-linking.

**EOS oxygenator membrane with phosphorylcholine coating**

Surface coatings based on phosphorylcholine (PC) were designed to provide an alternative to heparin-bound systems. Especially for patients suffering from heparin-induced thrombocytopenia (HIT), a surface coating with phosphorylcholine is a sensible option. The metabolic intermediate phosphorylcholine is antithrombotic, protein-resistant and antibacterial [5]. The "Phosphorylcholine Inert Surface in oxygenation" coating (PH.I.S.I.O) of the EOS oxygenator consists of physiologically inert material that imitates human endothelium. The hydrophilic phospholipid surface of this coating is designed to prevent activation of the coagulation system during contact with blood [6]. Phosphorylcholine is the polar head group of some phospholipids, between which there are negatively charged phosphate molecules, which in turn are bound to positively charged choline molecules [7].

**PLS oxygenator membrane with Bioline coating**

The BIOLINE coating utilises covalent bonds between heparin molecules and an albumin layer to create a hydrophilic and homogeneous surface. This form of surface coating promises to inhibit coagulation activation and potential thrombus formation [8][9].


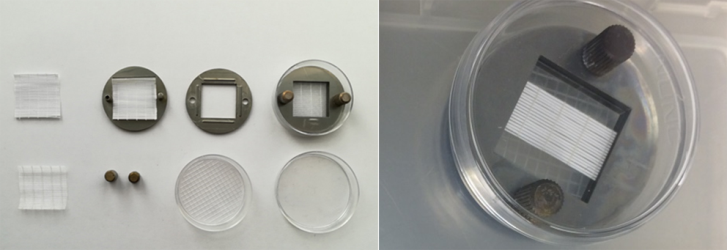


**Supplemental figure S1**. Membrane sample in stainless steel insert in petri dish. The sample consisted of 10 fibers (gas fibers or heat exchanger membranes) that were connected via wrap threads (dimension, 25 mm x 5 mm).

**
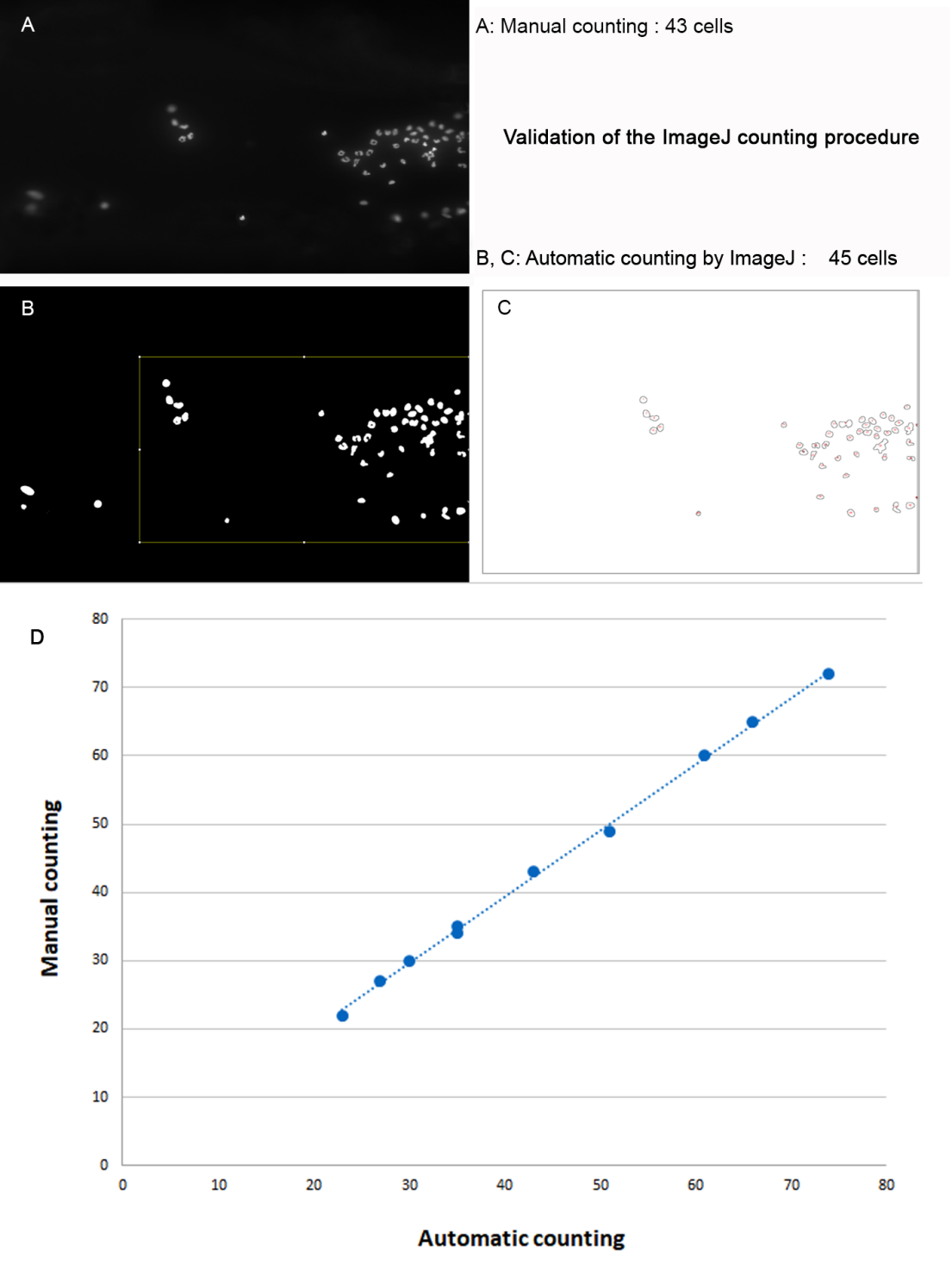
**

**Supplemental figure S2**. **Exemplary the validation of the automatic cell counting function.** For validation purposes, a preliminary study was carried out to determine whether there was a significant difference between the automatic cell counts (C) determined and those analysed by an experienced specialist (A). No significant difference was found between manual and automatic counting. Figure B visualises the region of interest (ROI) in ImageJ illustrated by the yellow box and the resulting detection and counting of cells. Correlation between manually and automatically counted nuclei (D).


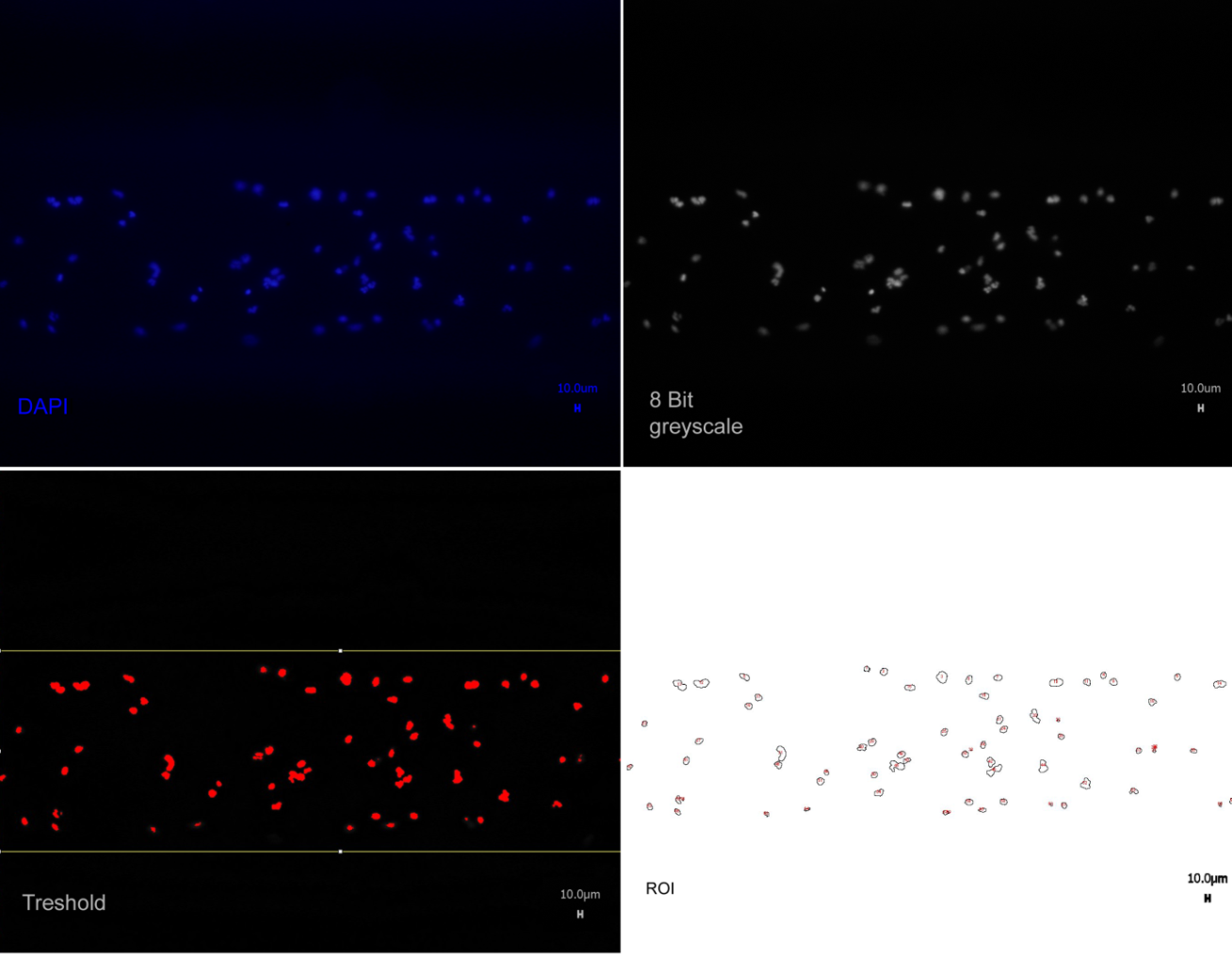


**Supplemental figure S3.** Time sequence of the automatic cell counting function of the image-processing programme ImageJ. Import of the DAPI-coloured original image in 40x magnification (top left) and conversion of the image information into an 8-bit Greyscale (top right). Following the definition of the detection limits (bottom left) and the region of interest (ROI) (bottom left), automated cell counting is performed.


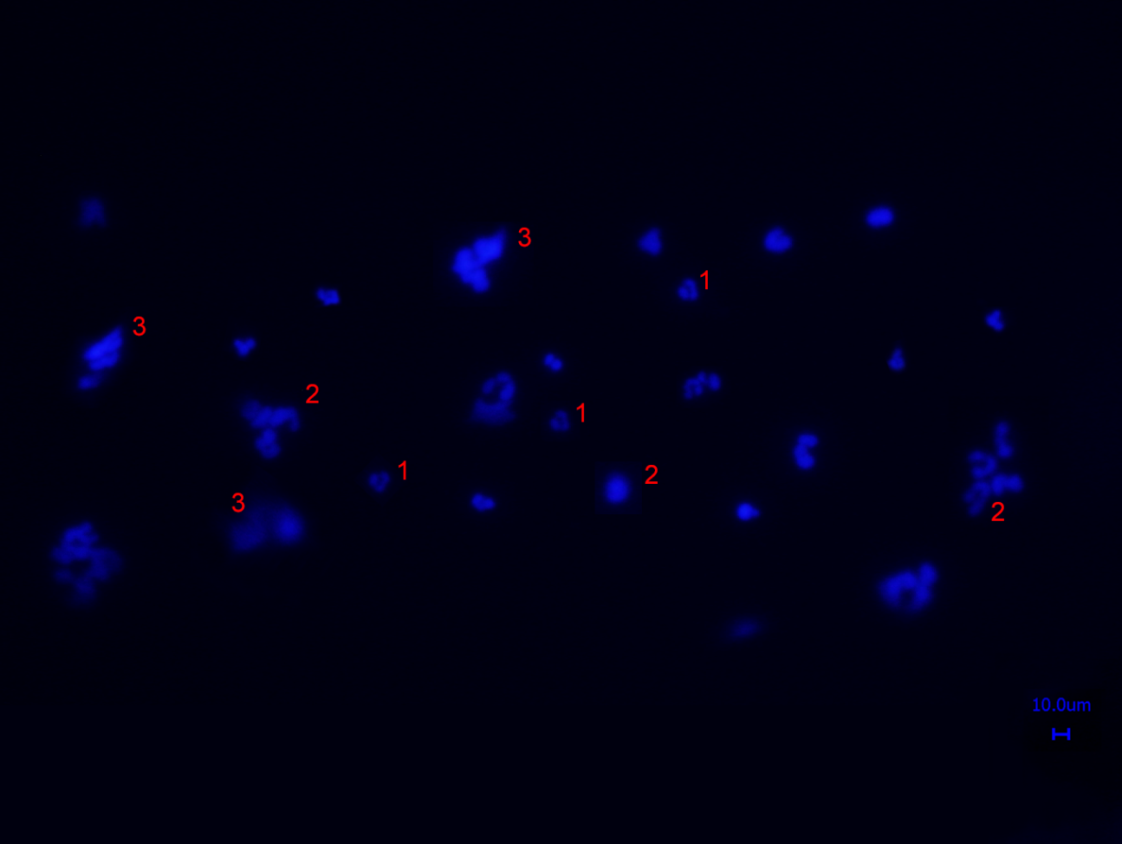


**Supplemental figure S4 (A) Uncoated-GF** (reference material). Classification of morphological changes: Granulocytes without any morphological changes (1). Swollen neutrophil granulocytes (2) of phase II and those that were about to rupture the plasma membrane in phase III or in which NETs were already detected (3).


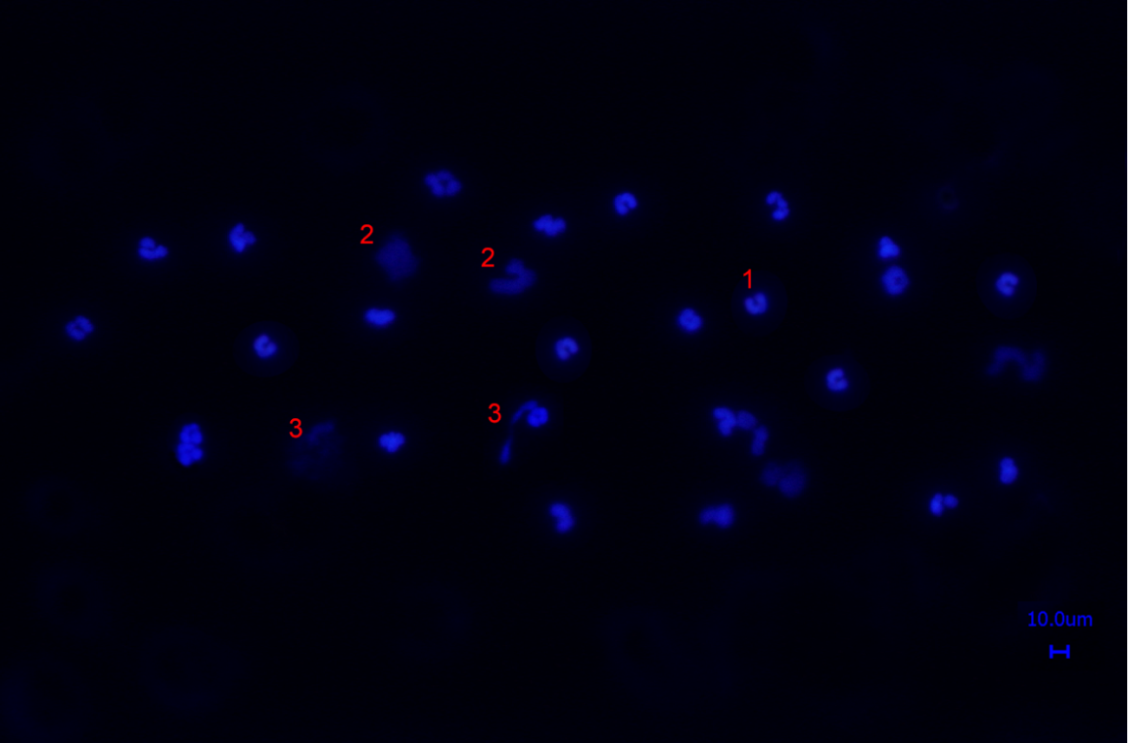


**Supplemental figure S4 (B) PLS-GF**. Classification of morphological changes: Granulocytes without any morphological changes (1). Swollen neutrophil granulocytes (2) of phase II and those that were about to rupture the plasma membrane in phase III or in which NETs were already detected (3).


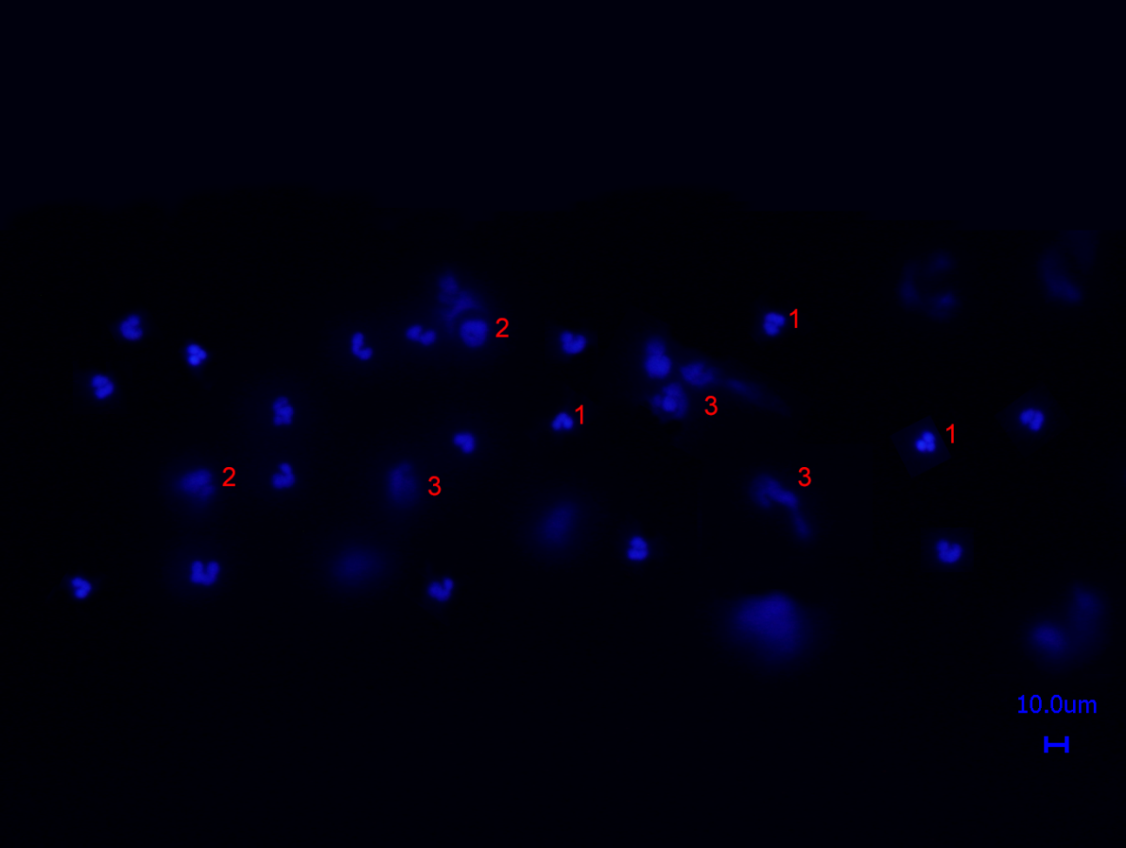


**Supplemental figure S4 (C) Hilite-GF.** Classification of morphological changes: Granulocytes without any morphological changes (1). Swollen neutrophil granulocytes (2) of phase II and those that were about to rupture the plasma membrane in phase III or in which NETs were already detected (3).


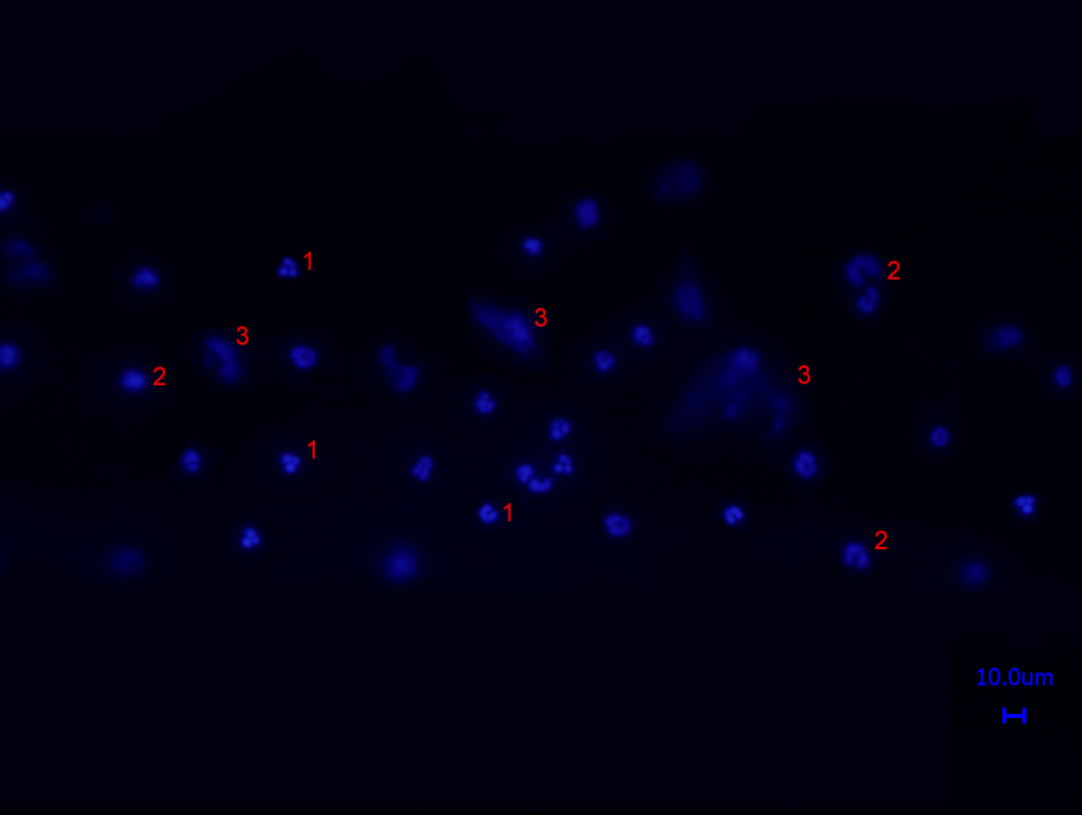


**Supplemental figure S4 (D) Nautilus-GF.** Classification of morphological changes: Granulocytes without any morphological changes (1). Swollen neutrophil granulocytes (2) of phase II and those that were about to rupture the plasma membrane in phase III or in which NETs were already detected (3).

 
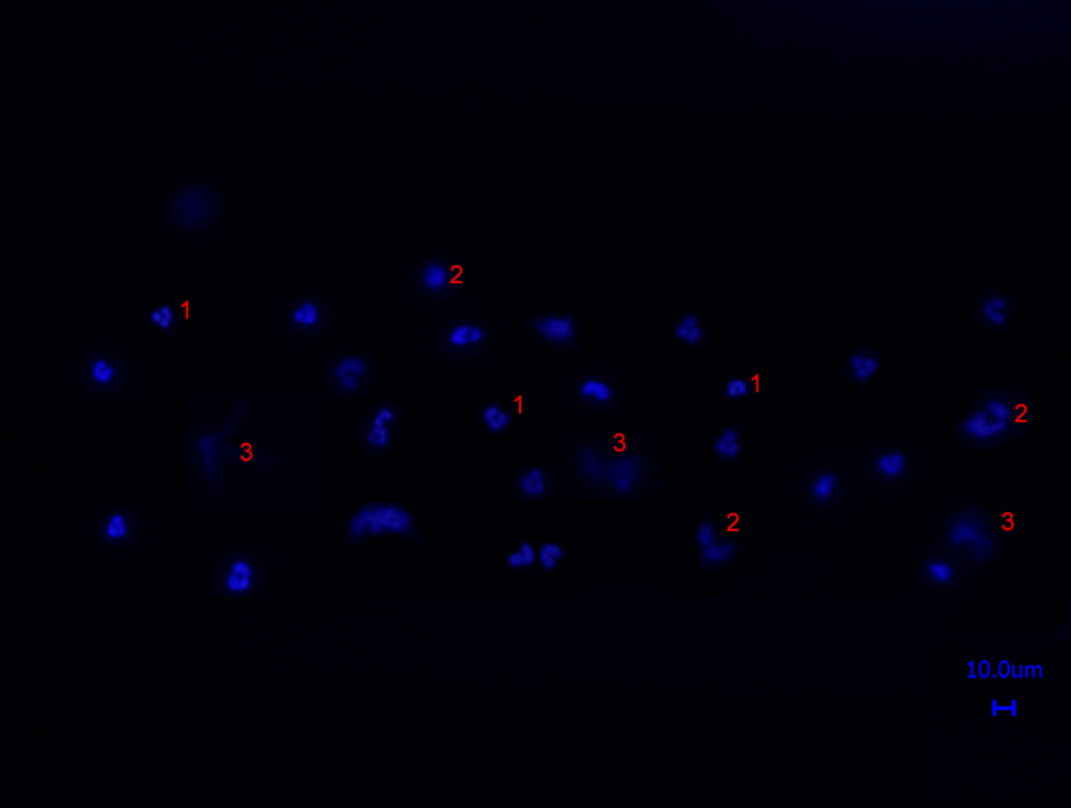


**Supplemental figure S4 (E) EOS-GF**. Classification of morphological changes: Granulocytes without any morphological changes (1). Swollen neutrophil granulocytes (2) of phase II and those that were about to rupture the plasma membrane in phase III or in which NETs were already detected (3).


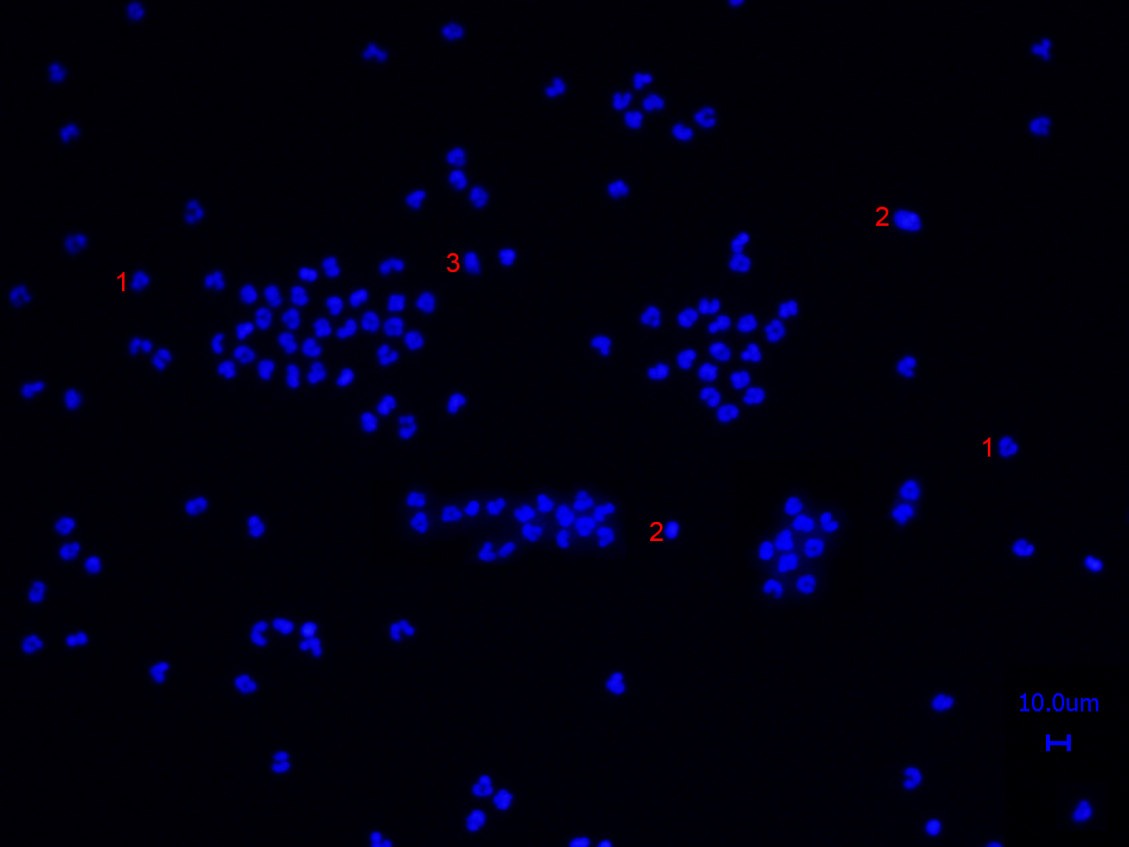


**Supplemental figure S4 (F).** Negative control of granulocytes on PLL-coated glass slides (80x magnification). Granulocytes without any morphological changes (1). Swollen neutrophil granulocytes (2) of phase II and those that were about to rupture the plasma membrane in phase III or in which NETs were already detected (3). Representative microscopic image from one blood donor (in total, 36 images from 6 blood donors),


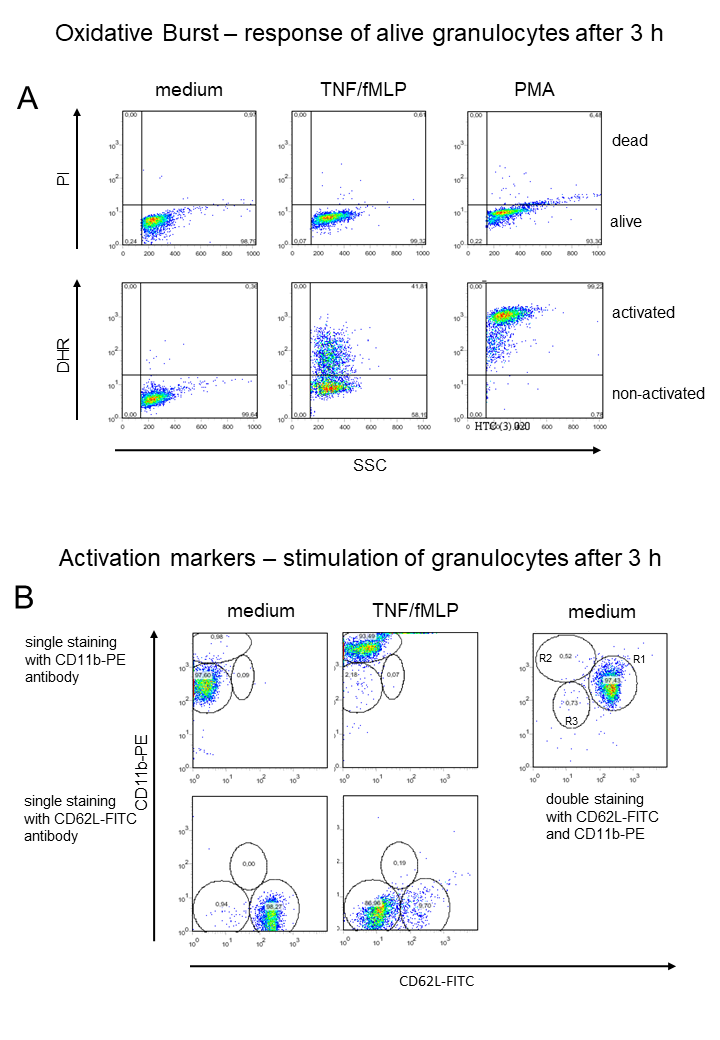


**Supplemental figure S5**. Quantification of the oxidative burst (A) and expression of activation markers (CD11b, CD62L) (B) using FACS analysis. Isolated granulocytes were treated with medium, TNF/fMLP or PMA as described in the method section. (A) Propidiumiodid (PI) visualized dead (PI-positive) and alive (PI-negative) cells. PI-negative cells were selected to detect ROS-producing cells (DHR-positive, activated). The proportion of alive cells that were DHR-positive were analyzed. (B) Single antibody staining was used to compensate the FACS channels. Granulocytes in medium presented moderate CD11b-PE (high CD62L-FITC) fluorescence intensity, while stimulation witSlh TNF/fMLP increased (decreased) the signals. Cells in the supernatants were stained with both antibodies and illustrated in a dotplot of CD62L-FITC vs CD11b-PE (right). Gated cell populations are defined as non-activated (R1, CD62L+/CD11b-) and activated (R2, CD62L-/CD11b+; R3, CD62L-/CD11b-).

**Implementation of the PMN-isolation of granulocytes**

Using a 20-G needle and filled into three 7.5 mL Li-heparin blood tubes (S-Monovette^®^ Lithium-heparin, 16 IU/ml blood, Sarstedt AG & Co, Nuremberg). The donors’ blood then rested for 20 min at 10 rpm on a tube roller (RS-TR10 Phoenix Instrument GmbH, Karlsdorf-Neuthard). Granulocytes were isolated by double density gradient centrifugation: 3 mL of Leuko Spin Medium, 3 mL of PBMC Spin Medium (pluriSelect Life Science, Leipzig) and 3 mL of heparinised blood gently deposited on top, taking care to avoid mixing according to manufacturer´s instructions. After centrifugation (1,000x g, room temperature, 30 min), the plasma and the PBMC Spin Medium layers were carefully discarded. The granulocytes were removed and resuspended in Hepes-Tyrode´s buffer containing 0.5 % bovine serum albumin (BSA), calcium (2 mM) and magnesium (1 mM) (HTP-BSA-Ca/Mg). Isolated PMNs from each volunteer were incubated with all test materials in six independent experiments. Cell count was determined in 10 randomly selected and non-overlapping microscopic images (magnification, 40x) from each sample (10 images/sample, 6 blood donors, 5 different GF-coatings = 300 images). For glas slides, 5 randomly selected images from each blood donor was analyzed (= 30 images). NETotic nuclei were identified on 180 images (magnification, 80x) from GF-samples (6 images/sample, 6 blood donors, 5 different GF-coatings) and on 36 images from glas slides (6 images per blood donor).

**Implementation of the material-induced activation of granulocytes – FACS analysis**

Oxidative burst: Three aliquots of the supernatant (each 100 µl with 4x10^5^ cells) were diluted in 2 mL phosphate-buffered saline (PBS) and centrifuged (250x g, RT, 10 min). The cell pellet was resuspended in PBS (1 mL) and 100 µl of dihydrorhodamine/SNARF (DHR/CargoxySNARF-1, 1 mM/10 µM; D632/C1271; Invitrogen, California, USA) was added for the burst experiments. The non-fluorescent DHR was oxidised intracellulary to green fluorescent rhodamine 123. The amount of rhodamine 123 was proportional to generated ROS. After a 10 min incubation at 37 °C, the first aliquot was spiked with 10 µl tumour necrosis factor (TNF-α, Thermo Fisher Scientific, 1 µg/mL in PBS) and 10 µl N-formyl-L-methionyl-L-leucyl-phenylalanine (fMLP, F3506, Sigma-Aldrich, St. Louis, USA; 1 mM in dimethylformamid, DMF), the second aliquot was spiked with 10 µl PMA (1 mM in DMF), and the third aliquot was spiked with 10 µl PBS. After a further 20 min incubation at 37 °C, 10 µl propidium iodide (PI, P1304MP Invitrogen, 1 mg/mL) was added. Dead cells were identified by increased PI fluorescence (emission above 600 nm) and lack of esterase activity determined based on SNARF1-related orange fluorescence. SNARF1/AM (non-fluorescent) was cleaved in vital leukocytes by esterases to SNARF1. Only vital cells (PI-negative) were selected to detect ROS-producing cells (DHR-positive) (Figure S3, supplementary material).

Expression of activation markers: Two aliquots of the supernatant (each 5x10^5^ cells) were diluted in 2 mL phosphate-buffered saline (PBS-Ca/Mg) and centrifuged (250x g, RT, 10 min). The cell pellet was resuspended in 100 µl PBS-Ca/Mg (1 mL) supplemented with monoclonal primary antibodies (antihuman CD62L-FITC, antihuman CD11b-PE; 304804/301306, Biolegend, San Diego, USA; each 5 µl) and incubated for 15 min (4°C, darkness). After addition of 2 mL PBS-Ca/Mg and centrifugation (290x g, RT, 5 min), the cell pellet was resuspended in 200 µl PBS and fixed with 1 mL BD-lysis Reagent (1:10, BecktonDickenson Biosciences, Heidelberg) (10 min, RT, darkness). After dilution with 2 mL PBS, cells were centrifuged (290g, 5 min, RT) and pellet was resuspended in 200 µL PBS. As a positive control, isolated granulocytes were stimulated with TNF/fMLP (as described for the oxidative burst) and stained with the antibodies. Non-stimulated cells were used as a negative control.

A FACS Calibur™ flow cytometer (BD corporate, Franklin Lakes, NJ, USA) and CellQuest Pro software™ (Version 5.2, BD corporate, Franklin Lakes, NJ, USA) was used. Granulocytes were identified by their typical forward (FSC) and side scatter (SSC) light patterns and their SNARF-1 fluorescence. The activation status was analyzed using a dotplot (CD11b-PE vs CD62L-FITC). Non-stimulated granulocytes were identified with a positive signal for CD62L and a moderate signal for CD11b. After activation with TNF/fMLP, the majority of PMNs expressed a higher signal for CD11b and CD62L was reduced.

References

**1**. Coleman RW, Clowes AW, George JN, Hirsh J, Marder V. Hemostasis and Thrombosis. Basic Principles and Clinical Practice, Fourth Edition. Robert W. Colman, Jack Hirsh, Victor J. Marder, Alexander W. Clowes, and James N. George, eds. Philadelphia, PA: Lippincott Williams & Wilkins, 2001, 1578 pp., $249.00, hardcover. ISBN 0-7817-1455-9. Clinical Chemistry. 2003; 49:345-a-346. doi: 10.1373/49.2.345/-a.

**2**. Charef S, Tapon-Bretaudière J, Fischer AM, Pflüger F, Jozefowicz M, Labarre D. Heparin-like functionalized polymer surfaces: discrimination between catalytic and adsorption processes during the course of thrombin inhibition. Biomaterials. 1996; 17:903–12. doi: 10.1016/0142-9612(96)83286-8 PMID: 8718936.

**3**. Silver JH, Hart AP, Williams EC, Cooper SL, Charef S, Labarre D, et al. Anticoagulant effects of sulphonated polyurethanes. Biomaterials. 1992; 13:339–44. doi: 10.1016/0142-9612(92)90037-o PMID: 1610956.

**4**. Grasel TG, Cooper SL. Properties and biological interactions of polyurethane anionomers: effect of sulfonate incorporation. J Biomed Mater Res. 1989; 23:311–38. doi: 10.1002/jbm.820230304 PMID: 2715157.

**5**. Tanzi MC. Bioactive technologies for hemocompatibility. Expert Rev Med Devices. 2005; 2:473–92. doi: 10.1586/17434440.2.4.473 PMID: 16293086.

**6**. Vertellus Biomaterials. PC coatings improve the biocompatibility of medical devices. 2024 [cited 1 Jul 2024]. Available from: https://www.medicaldevice-network.com/contractors/material-solutions/vertellus-bio/.

**7**. Willers A, Arens J, Mariani S, Pels H, Maessen JG, Hackeng TM, et al. New Trends, Advantages and Disadvantages in Anticoagulation and Coating Methods Used in Extracorporeal Life Support Devices. Membranes (Basel). 2021; 11. Epub 2021/08/12. doi: 10.3390/membranes11080617 PMID: 34436380.

**8**. Brash JL, Uniyal S. Dependence of albumin–fibrinogen simple and competitive adsorption on surface properties of biomaterials. J polym sci., C Polym symp. 1979; 66:377–89. doi: 10.1002/polc.5070660135.

**9**. Chuang HY, King WF, Mason RG. Interaction of plasma proteins with artificial surfaces: protein adsorption isotherms. J Lab Clin Med. 1978; 92:483–96
